# A heterogeneous thermal environment enables remarkable behavioral thermoregulation in *Uta stansburiana*

**DOI:** 10.1002/ece3.1141

**Published:** 2014-08-05

**Authors:** Maria Goller, Franz Goller, Susannah S French

**Affiliations:** 1 Department of Biology, Utah State UniversityLogan, Utah, 84322-5305; 2 Department of Biology, University of UtahSalt Lake City, Utah, 84112-0840

**Keywords:** Side-blotched lizard, thermal microhabitat, thermal preference, *Uta stansburiana*

## Abstract

Ectotherms can attain preferred body temperatures by selecting specific temperature microhabitats within a varied thermal environment. The side-blotched lizard, *Uta stansburiana* may employ microhabitat selection to thermoregulate behaviorally. It is unknown to what degree habitat structural complexity provides thermal microhabitats for thermoregulation. Thermal microhabitat structure, lizard temperature, and substrate preference were simultaneously evaluated using thermal imaging. A broad range of microhabitat temperatures was available (mean range of 11°C within 1–2 m^2^) while mean lizard temperature was between 36°C and 38°C. Lizards selected sites that differed significantly from the mean environmental temperature, indicating behavioral thermoregulation, and maintained a temperature significantly above that of their perch (mean difference of 2.6°C). *Uta*'s thermoregulatory potential within a complex thermal microhabitat structure suggests that a warming trend may prove advantageous, rather than detrimental for this population.

## Introduction

Ectotherms use behavioral (active) thermoregulation extensively to allow optimization of physiological processes (Huey and Stevenson 1979; Dzialowski and O'Connor 2001). Thermoregulation is a highly complex problem because different physiological processes and behaviors achieve performance optima at different temperatures (Huey and Slatkin 1976; Huey and Stevenson 1979; Angilletta et al. 2009). Lizards generally thermoregulate by choosing when to be active throughout the day and season (Stevenson 1985; Adolph and Porter 1993), and shuttling between microhabitats of differing temperatures (Waldschmidt 1980; Stevenson 1985; Adolph 1990; Gvozdik 2002).

Many ectotherms follow a daily cycle of thermal microhabitat preference (Hutchinson and Maness 1979; Stevenson 1985). The potential for behavioral thermoregulation is therefore limited by the available thermal niches, and the degree of microhabitat heterogeneity determines the potential for regulating body temperatures (Soulé 1963; Huey and Slatkin 1976; Stevenson 1985; Adolph 1990; Hertz et al. 1993; Smith and Ballinger 2001; Sartorious et al. 2002; Blouin-Demers and Nadeau 2005). Greater variability in habitat constitutes greater potential for precise regulation of preferred temperatures. More opportunity for thermoregulation contributes to the increased fitness of organisms in a complex habitat (Alexander and Whitford 1968; Fox 1978; Huey 1991; Smith and Ballinger 2001), as higher quality thermal environments decrease cost, while increasing precision, of regulation (Blouin-Demers and Nadeau 2005). Availability of a variety of environmental temperatures facilitates thermal choice in the context of other competing needs concerning microstructure of the habitat. Despite the importance of thermal microhabitats there is little information on thermal niche mosaics for any ectotherm, and, thus, it is also not known how the microhabitat can influence the ability to behaviorally thermoregulate.

**Figure 1:**
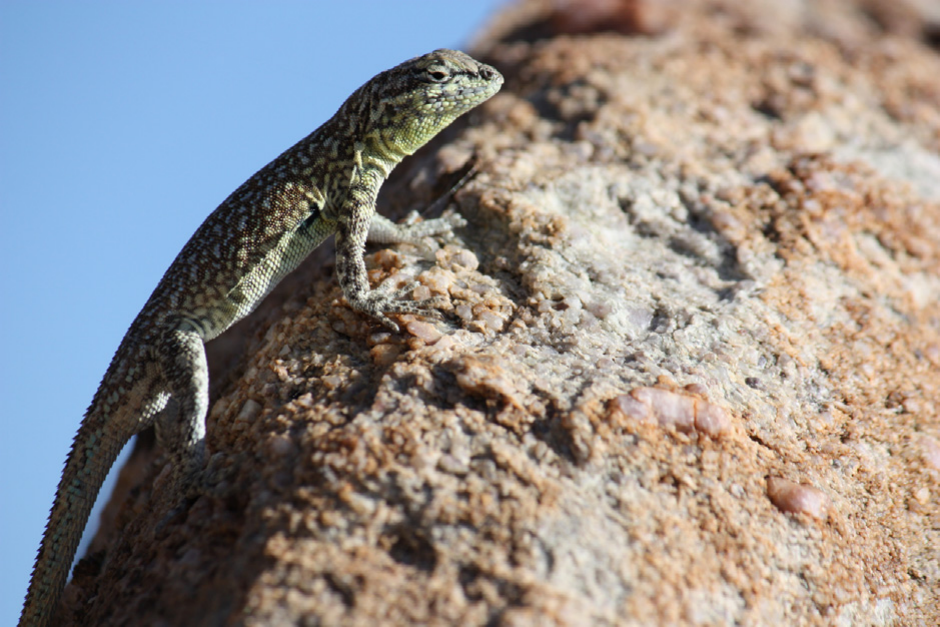
The study organism, *Uta stansburiana* (photograph by M. G.).

The side-blotched lizard, *Uta stansburiana* (Baird & Girard 1852; Fig.1), is a small, diurnal iguanid lizard found in desert regions across western North America (e.g., Irwin 1965; Evans 1967; Parker and Pianka 1975). This species is very common and inhabits a wide range of habitats (Nussbaum and Diller 1967; Tinkle 1967). *Uta stansburiana* are sit-and-wait predators (e.g., Parker and Pianka 1975; Waldschmidt and Tracy 1983), and presumably have time to regulate precisely for preferred body temperature.

Here we used thermography to measure the thermal complexity of *Uta* microhabitats during the hottest part of the year (July and August). This work illustrates a novel method that provides opportunities for more fine-tuned hypothesis testing of behavioral thermoregulation. In the current study, we determined temperature microhabitat structure surrounding lizards as well as their surface temperatures in a heterogeneous environment to explore to what degree lizards are able to use temperature differences within their microhabitat to behaviorally thermoregulate. A detailed quantitative evaluation of thermal habitat and the interaction between lizard and environmental temperatures should enable predictions for how climate change might affect this population. This study therefore can serve as a model for investigation of the thermal ecology of diverse organisms.

## Materials and Methods

### Study organism and sampling

**Figure 2:**
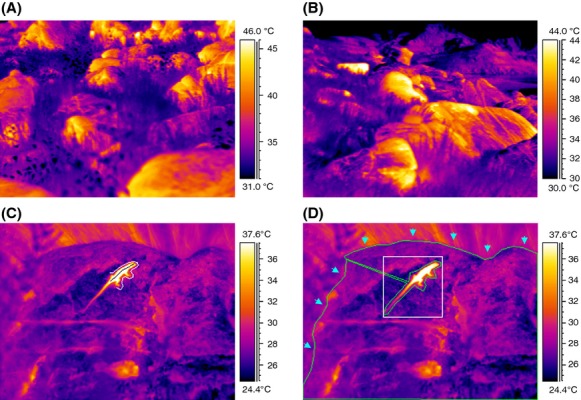
Thermograms of temperature microhabitats in the overall landscape (A–B) and analysis of lizard and environmental temperature (C–D). Measurement of lizard average temperature (line) and perch temperature (outline) is seen in (C). (D) Determination of maximum lizard temperature (box) and environmental maximum and minimum from the entire visible substrate available to the lizard. Only the rock surface (arrows indicate rock outline) was included in environmental analyses.

*Uta stansburiana* is a small species, with a snout-vent length of 50 mm and mass of 3.5–4 g. Populations on Utah's Antelope Island, located in the Great Salt Lake, are small and widely dispersed. The study population was on the northwestern tip of the island, around Buffalo Point (41.04° latitude, −112.27° longitude). Habitat consisted of isolated boulders of variable size, surrounded by grass, small bushes, and sunflowers (Fig.2A and B). Individual lizards inhabited boulders that were varying distances (10 cm to several m) apart. Different areas of the habitat were visited on subsequent days, and lizards (*n* = 23) were located and filmed on 19 days over a 2-month period, 9 July through 22 August 2011, as they were encountered. Unless an individual had been filmed earlier, the next encountered lizard was chosen as the subject. Individuals were filmed at various times of day, ranging from 7:30 to 19:30, a time frame covering the full period of lizard activity.

Camera measurements allowed noninvasive observations of thermal behavior. Although lizards were not captured and marked, the selection of locations for recording ensured that repeat sampling of the same individual was avoided. There was little overlap of territories as home ranges were small and individuals moved only short distances. No intraspecific interactions were observed.

### Field sampling

Lizard thermal preference data were collected by measuring environmental and lizard temperatures simultaneously with an infrared camera (ThermaCAM® S65HS; FLIR Systems, Wilsonville, OR, USA). A lizard was approached and either filmed at 10 frames/sec or photographed at 0.1 frames/sec for varying lengths of time (minimum of 10–25 min, up to several hours). Environment around the lizard was included in each frame, such that available thermal niches could be assessed.

Overall accuracy for the camera is specified as ±2°C for absolute temperature measurements. The important aspect for this study, however, is the relative temperature difference between pixels within single images, and the resolution for this aspect is 0.1°C. Although infrared cameras measure surface temperature, core body temperature does not deviate by more than 1 ± 1°C in small lizards (Jones & Avery 1989; Carretero 2008; Luna & Font 2013).

### Image analyses

Images were analyzed manually with ThermaCAM Researcher Professional 2.8 SR-1 (FLIR Systems) and various temperature measurements were made in each frame: (1) environmental (substrate) maximum and minimum temperatures, (2) lizard mean and maximum temperatures, (3) range of lizard temperature, and (4) temperature of the rock section upon which the lizard was sitting (perch temperature). Each frame consists of 32,000 individual temperature pixels, giving a high-resolution image of all microhabitat temperatures. Measurements from a total of 7390 images were used in analysis. Environmental temperatures were measured by using the “draw” tool to enclose the entire visible substrate, excluding the lizard and other features (vegetation, sky; Fig.2D). Images quantified thermal complexity of an individual's territory by indicating all existing temperatures within microhabitats. The size of the focal area within an image varied (100–5200 cm^2^; when assuming depicted lizard length of 50 mm) due to differences in size of and distance from the rock substrate.

Mean lizard temperatures were determined with the use of a line drawn down the center of the lizard, from snout to vent (Fig.2C). Lizard maximum temperature was found by creating a box incorporating the entire lizard (if lizard temperature was greater than that of perch; Fig.2D). If the lizard was cooler than the substrate, the lizard mean line was reused for determining lizard maximum temperature. Perch temperatures were found by drawing a line around the lizard's torso and head (Fig.2C), excluding substrate beneath the tail.

### Analyses

**Figure 3:**
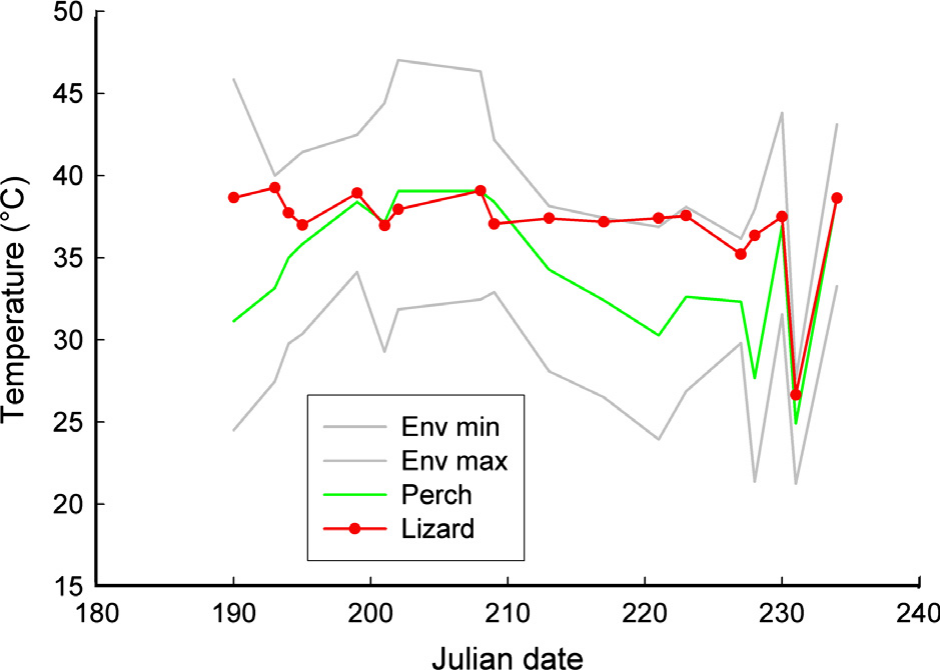
Changes in environmental (minimum, maximum, perch) and mean lizard body temperatures across the study period. There were no significant trends within July or August. As environmental temperatures fluctuated, lizard temperature remained constant (around 37°). Perch temperatures were above lizard temperature only at the end of July.

Environmental temperatures (minimum and maximum) between July and August (Fig.3) and mean daytime ambient air temperatures remained fairly constant (around 31°C). Temperature data across days were subsequently pooled for analyses.

A subset of 140 frames (haphazardly chosen across the entire sampling period) was selected across individuals and time of day. This subset was used to determine mean environmental temperature and standard deviation to assess environmental variability in more detail. These measurements – in addition to minimum and maximum temperature – were used to test whether lizards select perch temperature directly or indirectly. In the latter case, mean perch temperature should be randomly distributed around the mean environmental temperature. To account for variability across different recordings, we calculated a *z*-score using the standard deviation in pixel measurements for each frame. To do this, the difference between perch temperature and mean environmental temperature was expressed relative to the standard deviation (standardized), where a score of 1 was a difference of one standard deviation and a score of 0 was no difference between perch temperature and mean environmental temperature.

Statistical analyses are specified throughout the results. Linear regression statistics were calculated in Sigma Plot (v. 8.2) (Systat software, San Jose, California) and Kolmogorov–Smirnov tests were run in SPSS (IBM SPSS, Armonk, New York). We performed three tests to ascertain whether lizard perch temperature is different from the mean environmental temperature. We used a *t*-test to test whether the slope of the regression differed from a theoretical slope of 1. The *t*-value was computed as *t* = (slope − theoretical slope)/standard error of regression with *n* − 2 degrees of freedom. To test whether two independent regressions have a different slope, we calculated 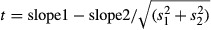 with *n*_1_ + *n*_2_ − 4 degrees of freedom. To test whether perch temperature is different from mean environmental temperature, we calculated *z*-scores to pool all differences irrespective of the absolute temperature and to account for the differing variance in environmental temperature, and compared the distribution to a normal distribution.

## Results

### Thermal microhabitat structural complexity

**Figure 4:**
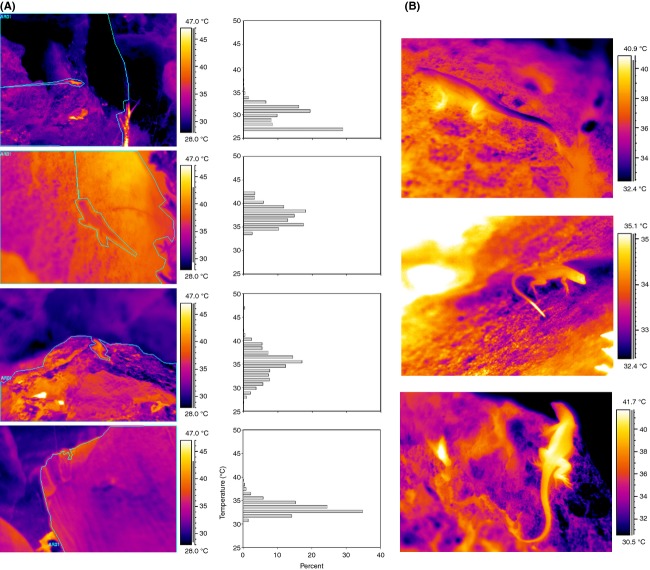
(A) The thermal diversity of microhabitats and corresponding histograms of available substrate temperatures surrounding different individuals. (B) Thermograms of the variability in lizard body temperature and preferred substrate temperatures. Varying temperatures across an individual could be achieved by increasing or decreasing contact between body and substrate.

**Figure 5:**
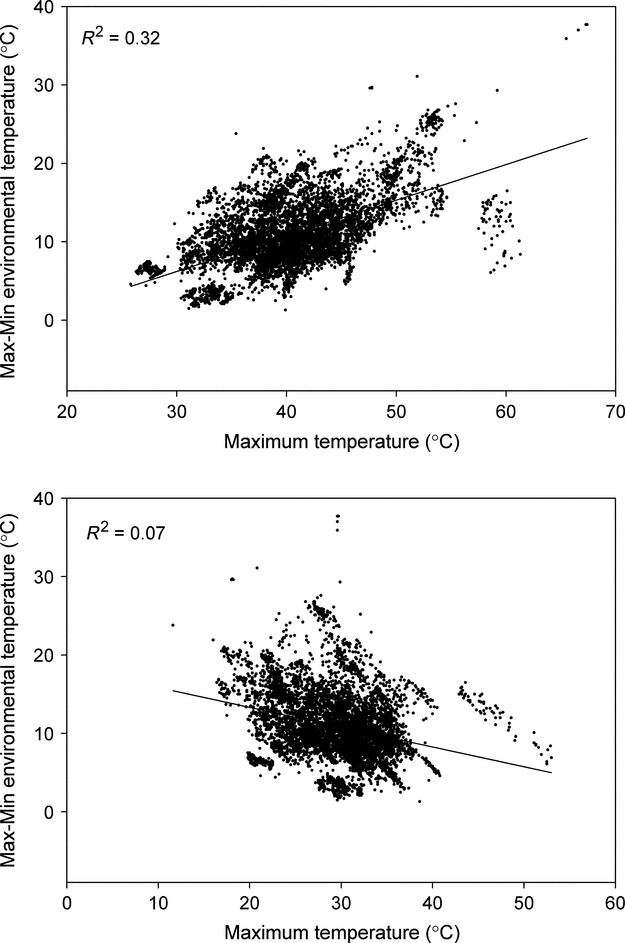
Maximum and minimum environmental temperatures plotted against the range of environmental (available) temperature. Range increased with maximum temperature (Range = −7.4 + 0.45 × Max T; *F* = 3529; *P* < 0.0001; *R*^2^ = 0.32) and decreased with increasing minimum temperature (Range = 18.36 − 0.25 × Min T; *F* = 579.0; *P* < 0.0001; *R*^2^ = 0.07). Change in maximum temperature explained more of the observed variation in range than did change in minimum temperature.

Lizards were found in a landscape characterized by high thermal heterogeneity – a conglomeration of isolated rock “heat islands” separated by cooler vegetation (Fig.2A and B). The entire habitat had a broad temperature range, generally from 25°C to 48°C, and even single boulders encompassed many different thermal microhabitats (Fig.4A). Each rock had a dynamic thermal microhabitat structure shaped by surrounding vegetation and irregularities in the rock's surface. Thermal variability was highly dependent on the presence or absence of direct solar radiation. The range of microhabitat temperatures at individual sites was as small as 1.3 or as broad as 37.7°C at any given time, and the mean temperature range (±1 SD) was 10.93 ± 4.29°C. Maximum temperature ranged from 25°C to 60°C. Minimum temperatures ranged from 15°C to 55°C. Mean temperature range (i.e., max–min) is positively related to maximum environmental temperature and negatively with minimum environmental temperature (Fig.5). Maximum temperature explains more of the variation in range (32%) than minimum temperature (7%).

### Lizard thermoregulation

**Figure 6:**
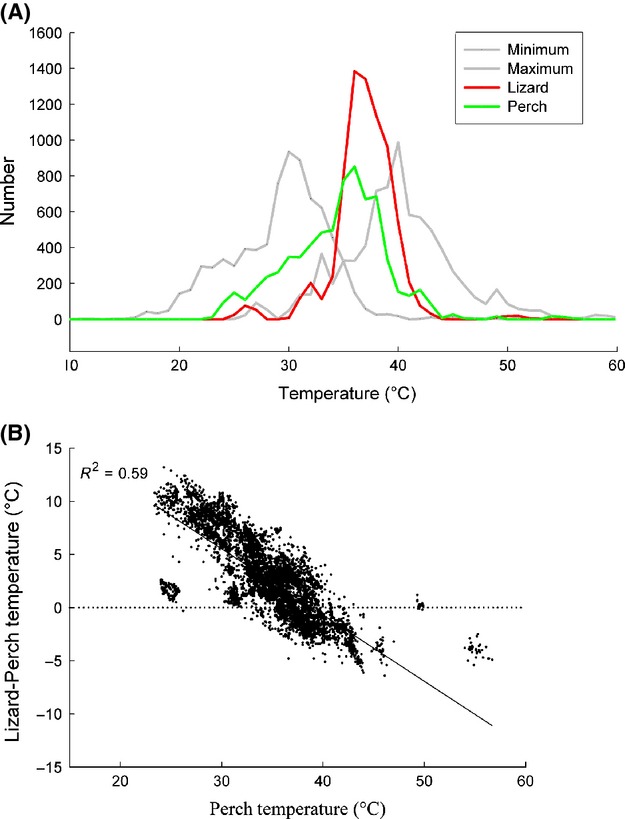
(A) Histogram showing the distribution of three environmental temperatures (perch, maximum, and minimum) and mean lizard body temperature. Lizard body temperature had the narrowest range, with a pronounced peak at 36–38°C, and was closer to maximum than to minimum temperature. (B) Perch temperature plotted against the difference between lizard mean body temperature and perch temperature. (Regression equation: Lizard – Perch *T* = 24.23 – 0.62 × Perch T; *F* = 9628; *P* < 0.0001).

Lizard body temperature has the potential to vary widely across a broad range of environmental temperatures at any given time (Fig.4B); however, overall lizard temperature remained fairly constant. Mean lizard temperature varied much less than environmental temperatures (Fig.6A). The most common maximum and minimum environmental temperatures were 40°C and 30°C, respectively. The mean pooled lizard temperature (across entire dataset) was 37.2 ± 2.9 °C, while mean maximum lizard temperature was 38.7 ± 3.1°C. The mean body temperature range encompassed solely by lizard midline analysis was 2.2°C (±1.32), though maximum individual range was 9°C. Temperature of the extremities differed more but was not measured systematically. Generally, lizard temperature was closer to maximum than minimum environmental temperature. The mean differences were 2.9 ± 3.8°C and 8.0 ± 4.0°C, respectively.

Lizard temperatures differed from perch temperature (Fig.6B) and as perch temperature increased, the mean of average lizard temperature approached perch temperature (Fig.6B, *R*^2^ = 0.59). Average lizard body temperature was above perch temperature at lower substrate temperatures and below at high perch temperature (above 37–38°C). Lizard mean temperature also approached perch temperature as average environmental temperature increased to 37°C (*R*^2^ = 0.429).

### Daily variation

**Figure 7:**
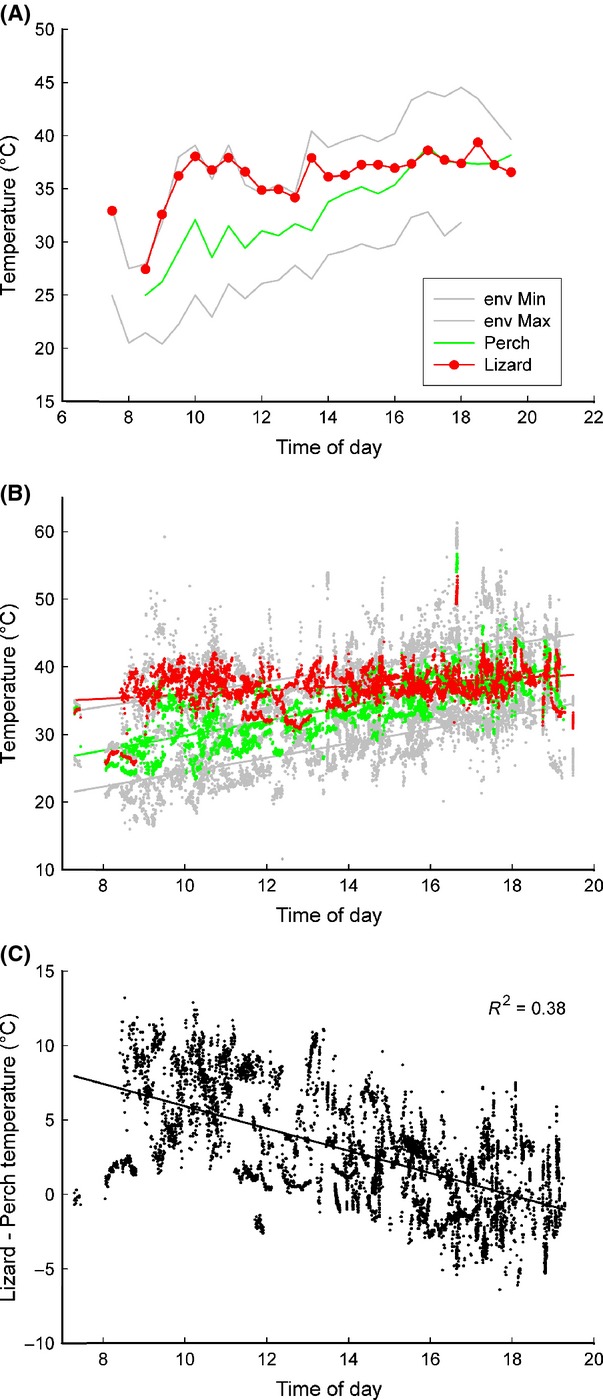
Temperature dynamics as a function of time of day, graphed as average temperature for every 30-min period (A) and all data points (B) and temperature difference (C). Slopes for temperatures (B) are as follows: Lizard T: slope = 0.3, *R*^2^ = 0.1; Min Env T: slope = 1.07; *R*^2^ = 0.51; Max Env T: slope = 0.93; *R*^2^ = 0.29; Perch T: slope = 1.1; *R*^2^ = 0.54. A small subset of values of lizard body temperature close to 50°C occurred over a 20-sec period, after which the lizard moved to deep shade. (C) Daily trends of the differences between lizard and perch temperature show that as environmental temperature increases over the course of the day perch temperature grows increasingly similar to body temperature.

When pooling temperature data by time of day, environmental temperatures increased throughout the day, with maximum temperature peaking at 18:00 (Fig.7A). Perch temperature also increased, stabilizing at 16:30. Lizard average temperature increased markedly throughout the morning and then remained relatively stable for the rest of the day. Slopes for environmental temperature variables all rose substantially over the course of the day, but that for lizard temperature remained flat (Fig.7B). The slope of lizard temperature was significantly different from the slopes of perch temperature, maximum and minimum environmental temperatures (*t*-test, in all three cases *P* < 0.0001). As expected from these different slopes, the difference between perch and lizard temperature decreased over the course of the day (*R*^2^ = 0.38; Figs.7C).

### Perch temperature

Perch temperature was close to, but significantly different from, mean environmental temperature. Perch temperature generally fell within the range of 35–38°C (Fig.6A). Three approaches were used to investigate whether perch temperature was a random selection or whether individual lizards chose specific temperatures. First, the mean difference between perch and environment was 0.36°C, and, although small, this difference was significant (paired *t*-test *P* = 0.02). Second, a linear regression of perch temperature over mean environmental temperature is highly significant (*P* < 0.0001) and the slope of 0.897 is significantly different from a slope of 1 (*t* = −3.233, *df* = 177; *P* < 0.005). Furthermore, this relationship explains 85.5% of the variation in perch temperature. Third, because perch temperature selection depends on the absolute environmental temperature and mean environmental temperature does not reflect variability, we calculated *z*-scores. The distribution of *z*-score perch temperature values differed significantly (Kolmogorov–Smirnov; *P* = 0.001) from a normal distribution (Fig.7C, indicating that perch temperature deviated systematically from mean environmental temperature, irrespective of the variance in absolute environmental temperatures.

### Positional switches

**Figure 8:**
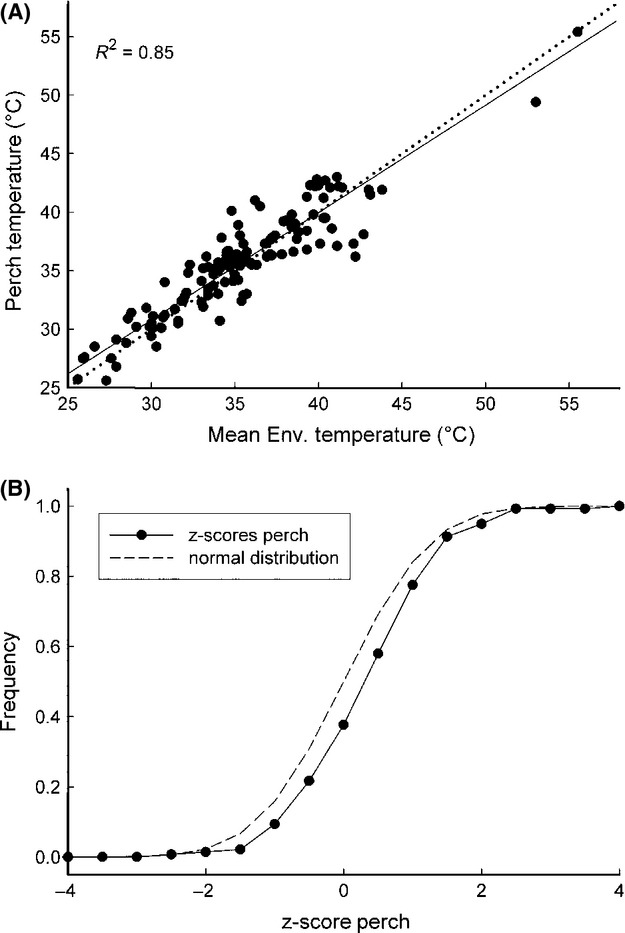
(A) Mean environmental temperature (of the subset) plotted against perch temperature, showing strong positive correlation. (Perch *T* = 3.97 + 0.897 × Env T; *F* = 803.0; *P* < 0.0001; slope is significant at *P* < 0.0001; intercept at *P* < 0.006; *R*^2^ = 0.85). (B) Distribution of the differences between perch and mean environmental temperature expressed as *z*-scores (of the subset) compared to a normal distribution. Distributions differed significantly, indicating selection of perch temperature (Kolmogorov–Smirnov, *D* = 0.174, *P* = 0.001).

**Figure 9:**
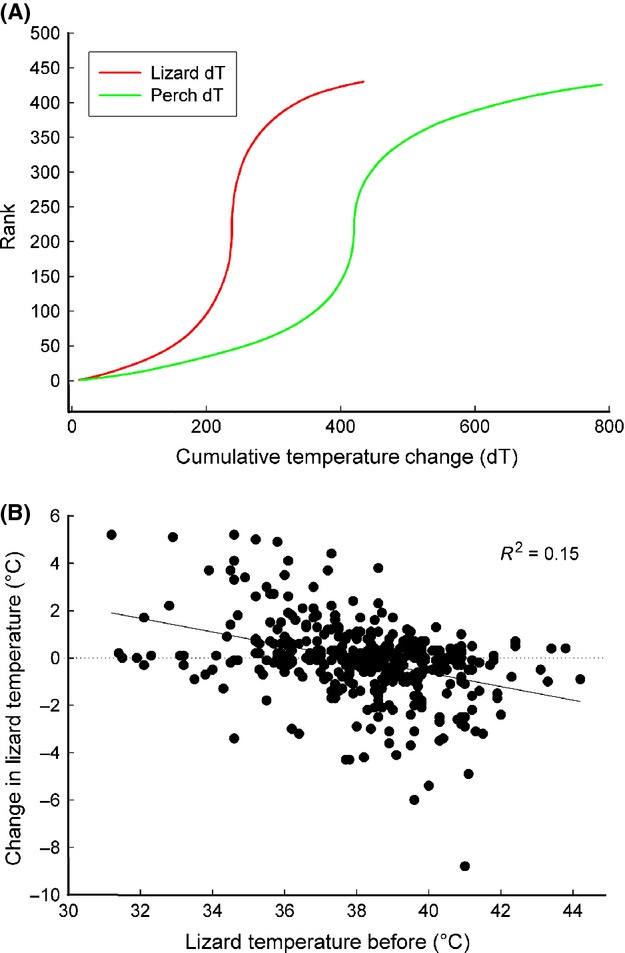
(A) Cumulative distributions of perch and lizard body temperatures illustrate the broader distribution of perch temperature change. (B) Lizard mean body temperature before the switch in position plotted against the change in body temperature (body temperature after – body temperature before).

Individuals spent substantial periods (from 10 to 100 min) sitting on one perch. Other activities included shifting position (moving slightly while remaining in the same microhabitat) or moving larger distances (onto a different portion of the same rock or onto a new rock altogether). A total of 434 switches were documented: 139 shifts and 295 moves. Perch and lizard average temperature were graphed as before/after for both categories (Fig.8). Time between “before” and “after” readings was 20 sec (for shifts) or several minutes (for moves). The cumulative distribution of change in perch temperature was broader than that of lizard body temperature (Fig.9A).

Lizards with a lower body temperature tended to grow warmer upon moving, and those with high body temperature colder (Fig.9B). The trend line illustrates a difference between before and after mean lizard temperature of ±2°C. This trend was particularly pronounced after moves (lizard temperature: slope = 0.801; *R*^2^ = 0.62; *P* < 0.0001; perch temperature: slope = 0.846; *R*^2^ = 0.72; *P* < 0.0001 were significantly different from a slope of one: *t*-test, *P* < 0.001 and *P* < 0.001) with the regression lines crossing the isothermal line at around 37°C. For switches the respective slopes were 0.977 and 0.970 and these were not significantly different from an isothermal slope of 1 (*t*-test, *P* = 0.2).

## Discussion

The goal of this study was to examine the thermal habitat complexity of a population of *Uta stansburiana* to assess its influence on behavioral thermoregulation. The results show a remarkably variable and complex environmental temperature landscape within the habitat both in space and over time. Within this variation in space and time, *Uta* effectively maintained a remarkably constant body temperature through behavioral thermoregulation. The detailed measurement of thermal microhabitat structure permits a more in-depth understanding of thermoregulatory behavior and thermal ecology. It therefore illustrates an important new approach to assessing potential impacts of global change on ectothermic organisms and allows for more specific hypotheses to be tested under ecologically relevant conditions.

### Thermoregulation in a heterogeneous environment

*Uta* showed effective behavioral thermoregulation and maintained an average body temperature of 37°C in highly variable thermal environments. The range in environmental temperature was mostly due to changes in maximum temperature, which suggests that patterns of solar radiation on the structured microhabitat are largely responsible for determining the range. Microhabitat heterogeneity produced by solar radiation and the three-dimensional structure of the habitat create the basis for effective behavioral thermoregulation by generating thermal diversity. A heterogeneous mosaic of small thermal microhabitats (high thermal quality) allows a species to achieve an optimal or preferred body temperature (Grant and Dunham 1988; Sartorious et al. 2002; Freidenburg and Skelly 2004; Monasterio et al. 2009). In the current study, individuals rarely engaged in shuttling behavior, suggesting that favorable microhabitats were readily available. Lizard body temperature was routinely significantly above or below perch temperature (Fig.6B) and individuals occasionally achieved a temperature exceeding that of the environmental maximum. The most likely explanation for these differential temperatures is that lizards, by being elevated above the substrate, could either gain more heat through solar radiation than the underlying rock or lose heat to a cooler air. This differential heat exchange may also have been facilitated by different emissivity/reflectivity of the lizard surface as compared to that of the microhabitat surface. However, further research is needed to precisely determine the respective contributions of these possibilities.

Individuals had remarkably stable daily body temperatures, with the exception of the early morning hour, despite a diurnal trend of increasing environmental temperatures and temperature fluctuations induced by weather changes. Lower morning body temperature is likely caused by lower availability of direct solar radiation to elevate temperature above that of the environment.

*Uta* demonstrated a clear preference for a narrow range of body temperature (36–38°C), which matches that found for both sexes in a laboratory study (Paranjpe et al. 2013). However, the microstructure in the habitat also resulted in diverging temperatures for different body parts. The temperature range across a lizard's core was up to 9°C within a single image, and extremities could deviate even further. Although a lizard could be exposed to differential levels of solar radiation and differing substrate temperatures (by sitting across multiple microhabitats), the size of the range is still surprising.

### Thermoregulation through perch selection

Within the range of available environmental temperatures, lizards selected a perch temperature that differed from the preferred body temperature (37°C) by only a few degrees. Although perch temperature was close to the mean temperature of the habitat, it still differed significantly. Active selection of perch temperature is suggested by several findings. First, temperature varied widely even within very small areas of substrate. Although mean environmental temperature was close to perch temperature, pixels with temperature readings close to the mean temperature were distributed across the monitored surface. Furthermore, perch temperature deviated by as much as ±6°C from mean environmental temperature. Without understanding the complex nature of the substrate, the close relationship between mean environmental and perch temperatures could be misconstrued as an indication of thermoconformity.

Further evidence for temperature selection can be derived from the lizards' movements between perches. The difference in perch temperature before and after a move was substantially greater than the change in lizard body temperature. The ability to maintain body temperature after the change in perch strongly suggests that thermoregulation played a role in selection of a new perch.

### Thermoregulatory trends

Díaz and Cabezas-Díaz (2004) found that microhabitat selection as a thermoregulatory strategy was more important in the summer. Temperature preferences may vary across seasons (Waldschmidt 1980), as in some *Sceloporus* species (Adolph 1990), and our population of *Uta* may show differing patterns of thermoregulation, or even acclimation, in other seasons with fluctuating air temperature. Our study illustrates that a high degree of thermal heterogeneity is driven by radiative exchange, not by air temperature, and it is therefore not surprising that no correlation was found between air temperature and body temperature in *Uta* (Soulé 1963), as found in tropical *Anolis* species (Huey and Webster 1975).

### Implications for climate change

A result of climate change will be greater variation and/or an increase in temperature across the range of *Uta stansburiana*. Although an increase in several degrees will probably provide a more optimal thermal environment for temperate species (Weatherhead et al. 2011), it will also increase the chance of overheating (Kearney et al. 2009), and rising temperatures may render habitats with less thermal heterogeneity unsuitable for *Uta*. An increase in temperature may not be detrimental to the study population. Higher thermal microhabitat diversity is important as it may allow behavioral thermoregulation to a preferred temperature in varying temperature conditions. Ability to thermoregulate by moving into shaded microhabitats can be an important buffer of climate change (Kearney et al. 2009), and complex habitats provide shade more reliably.

During July and August, individuals preferred substrate temperatures close to the maximum environmental temperature. Cooler microhabitats were available at all times but were largely avoided. Thus, it appears that individuals of the study population will have microhabitats of preferred temperature, even if air temperature rises, and cooler areas could serve as retreats against overheating (Chamaillé-Jammes et al. 2006; Kearney et al. 2009).

Although this study focused only on the hottest months, it is relevant for assessing the potential impact of climate change. The period of highest environmental temperatures also poses the most severe thermal challenges (Vickers et al. 2011), and warming during other seasons may be beneficial (Zani 2008). Sinervo et al. (2010) proposed that thermoregulatory responses evolved by lizard species might not be sufficient when faced with rising temperatures. However, behavioral regulation for some species appears more flexible and efficient than does physiological change (Muñoz et al. 2014). Specific physiological and ecological needs may vary between populations, and thermal thresholds may exist for critical life stages (i.e., reproduction, embryonic development) that were not observed (Davis and Verbeek 1972; Huey et al. 2010; Sinervo et al. 2010). Air temperature and rate of maximum temperature change during the breeding season were correlated with extinction in Mexican lizard species (Sinervo et al. 2010). However, temperature increases during the reproductive season are unlikely to have a major impact on *Uta* (Paranjpe et al. 2013).

Furthermore, many temperate species concerned with staying warm may benefit from increases in temperature (Huey and Tewksbury 2009). Climate change has also already positively impacted some species (Huey et al. 2009) found in complex habitats (Huey and Tewksbury 2009). Other widely distributed species, such as the European common lizard (*Zootoca vivipara*), may exhibit greater habitat selectivity to avoid thermal challenges (Herczeg et al. 2006). Thus, greater opportunity for thermoregulation contributes to the increased fitness of organisms in a complex habitat (Alexander and Whitford 1968; Fox 1978; Huey 1991; Smith and Ballinger 2001; Blouin-Demers and Nadeau 2005). Mortality of *Uta stansburiana* has been linked to thermoregulatory ability (Zani 2008), with survivors being more selective in habitat choice (Tinkle 1967; Fox 1978). Our results on behavioral thermoregulation and microhabitat diversity suggest that climate change may prove beneficial to populations of *Uta stansburiana* found in areas of thermal microhabitat complexity (Clarke and Zani 2012), such as the one on Antelope Island.

This case study on a lizard indicates the potential for exploring the thermal ecology of ectotherms with this method that combines a more detailed assessment of the thermal microhabitat than was previously possible and its effect on thermoregulation of ectothermic organisms. As indicated here, thermography provides detailed knowledge of the thermal structure of the habitat which cannot be gained from a few point measurements. Additionally, the fairly good spatial resolution of thermal imaging systems makes this method useful for studying a broad size and thermal range of organisms and allows for capture of dynamic changes in environmental and body temperatures with fine-scale temporal resolution.

## References

[b1] Adolph SC (1990). Influence of behavioral thermoregulation on microhabitat use by two *Sceloporus* lizards. Ecology.

[b2] Adolph SC, Porter WP (1993). Temperature, activity, and lizard life histories. Am. Natur.

[b3] Alexander CE, Whitford WG (1968). Energy requirements of *Uta stansburiana*. Copeia.

[b4] Angilletta MJ, Sears MW, Pringle RM (2009). Spatial dynamics of lizard behavior: lizards shift microhabitats to construct nests with beneficial thermal properties. Ecology.

[b500] Baird SF, Girard C (1852). Reptiles. Howard Stansbury, exploration and survey of the valley of the Great Salt Lake of Utah, including a reconnaissance of a new route through the Rocky Mountains.

[b5] Blouin-Demers G, Nadeau P (2005). The cost-benefit model of thermoregulation does not predict lizard thermoregulatory behavior. Ecology.

[b6] Carretero MA (2008). Preferred temperatures of *Tarentola mauritanica* in spring. Acta Herpetol.

[b7] Chamaillé-Jammes S, Massot M, Aragón P, Clobert J (2006). Global warming and positive fitness response in mountain populations of common lizards *Lacerta vivipara*. Glob. Change Biol.

[b8] Clarke DN, Zani PA (2012). Effects of night-time warming on temperate ectotherm reproduction: potential fitness benefits of climate change for side-blotched lizards. J. Exp. Biol.

[b9] Davis J, Verbeek NAM (1972). Habitat preferences and the distribution of *Uta stansburiana* and *Sceloporus occidentalis* in coastal California. Copeia.

[b10] Díaz JA, Cabezas-Díaz S (2004). Seasonal variation in the contribution of different behavioural mechanisms to lizard thermoregulation. Funct. Ecol.

[b11] Dzialowski EM, O'Connor MP (2001). Physiological control of warming and cooling during simulated shuttling and basking in lizards. Physiol. Biochem. Zool.

[b12] Evans KJ (1967). Observations on the daily emergence of *Coleonyx variegatus* and *Uta stansburiana*. Herpetologica.

[b13] Fox SF (1978). Natural selection on behavioral phenotypes of the lizard *Uta stansburiana*. Ecology.

[b14] Freidenburg LK, Skelly DK (2004). Microgeographical variation in thermal preference by an amphibian. Ecol. Lett.

[b15] Grant BW, Dunham AE (1988). Thermally imposed time constraints on the activity of the desert lizard *Sceloporus merriami*. Ecology.

[b16] Gvozdik L (2002). To heat or to save time? Thermoregulation in the lizard *Zootoca vivipara* (Squamata: Lacertidae) in different thermal environments along an altitudinal gradient. Can. J. Zool.

[b17] Herczeg G, Gonda A, Saarikivi J, Merila J (2006). Experimental support for the cost-benefit model of lizard thermoregulation. Behav. Ecol. Sociobiol.

[b18] Hertz PE, Huey RB, Stevenson RD (1993). Evaluating temperature regulation by field-active ectotherms: the fallacy of the inappropriate question. Am. Natur.

[b19] Huey RB (1991). Physiological consequences of habitat selection. Am. Natur.

[b20] Huey RB, Slatkin M (1976). Cost and benefits of lizard thermoregulation. Q. Rev. Biol.

[b21] Huey RB, Stevenson RD (1979). Integrating thermal physiology and ecology of ectotherms: a discussion of approaches. Am. Zool.

[b22] Huey RB, Tewksbury JJ (2009). Can behavior douse the fire of climate warming?. Proc. Natl Acad. Sci.

[b23] Huey RB, Webster TP (1975). Thermal biology of a solitary lizard: *Anolis marmoratus* of Guadeloupe, Lesser Antilles. Ecology.

[b24] Huey RB, Deutsch CA, Tewksbury JJ, Vitt LJ, Hertz PE, Perez HJA (2009). Why tropical forest lizards are vulnerable to climate warming. Proc. Biol. Sci.

[b25] Huey RB, Losos JB, Moritz C (2010). Are lizards toast?. Science.

[b26] Hutchinson VH, Maness JD (1979). The role of behavior in temperature acclimation and tolerance in ectotherms. Am. Zool.

[b27] Irwin LN (1965). Diel activity and social interaction of the lizard *Uta stansburiana stejnegeri*. Copeia.

[b501] Jones S, Avery RA (1989). The use of a pyroelectric vidicon infra-red camera to monitor the body temperatures of small terrestrial vertebrates. Funct. Ecol.

[b28] Kearney M, Shine R, Porter WP (2009). The potential for behavioral thermoregulation to buffer “cold-blooded” animals against climate warming. Proc. Natl. Acad. Sci. USA.

[b502] Luna S, Font E (2013). Use of an infrared thermographic camera to measure field body temperatures of small lacertid lizards. Herpetol. Rev.

[b29] Monasterio C, Salvador A, Iraeta P, Díaz JA (2009). The effects of thermal biology and refuge availability on the restricted distribution of an alpine lizard. J. Biogeogr.

[b30] Muñoz MM, Stimola MA, Algar AC, Conover A, Rodriguez AJ, Landestoy MA (2014). Evolutionary stasis and lability in thermal physiology in a group of tropical lizards. Proc. Biol. Sci.

[b31] Nussbaum RA, Diller LV (1967). The life history of the side-blotched lizard, *Uta stansburiana* Baird and Girard, in north-central Oregon. Northw. Sci.

[b32] Paranjpe DA, Bastiaans E, Patten A, Cooper RD, Sinervo B (2013). Evidence of maternal effects on temperature preference in side-blotched lizards: implications for evolutionary response to climate change. Ecol. Evol.

[b33] Parker WS, Pianka ER (1975). Comparative ecology of populations of the lizard *Uta stansburiana*. Copeia.

[b34] Sartorious SS, do Amaral JPS, Durtsche RD, Deen CM, Lutterschmidt WI (2002). Thermoregulatory accuracy, precision, and effectiveness in two sand-dwelling lizards under mild environmental conditions. Can. J. Zool.

[b35] Sinervo B, Mendez-de-la-Cruz F, Miles DB, Heulin B, Bastiaans E, Villagran-Santa Cruz M (2010). Erosion of lizard diversity by climate change and altered thermal niches. Science.

[b36] Smith GR, Ballinger RE (2001). The ecological consequences of habitat and microhabitat use in lizards: a review. Contemp. Herpetol.

[b37] Soulé M (1963). Aspects of thermoregulation in nine species of lizards from Baja California. Copeia.

[b38] Stevenson RD (1985). The relative importance of behavioral and physiological adjustments controlling body temperature in terrestrial ectotherms. Am. Natur.

[b39] Tinkle DW (1967). The life and demography of the side-blotched lizard, *Uta stansburiana*. Misc. Publications of Univ. of Michigan Museum of. Zoology.

[b40] Vickers M, Manicom C, Schwarzkopf L (2011). Extending the cost-benefit model of thermoregulation: high-temperature environments. Am. Natur.

[b41] Waldschmidt S (1980). Orientation to the sun by the iguanid lizards *Uta stansburiana* and *Sceloporus undulatus*: hourly and monthly variations. Copeia.

[b42] Waldschmidt SR, Tracy CR (1983). Interactions between a lizard and its thermal environment: implications for sprint performance and space utilization in the lizard *Uta stansburiana*. Ecology.

[b43] Weatherhead PJ, Sperry JH, Carfagno GLF, Blouin-Demers G (2011). Latitudinal variation in thermal ecology of North American rat-snakes and its implications for the effect of climate warming on snakes. J. Therm. Biol.

[b44] Zani PA (2008). Climate change trade-offs in the side-blotched lizard (*Uta stansburiana*): effects of growing-season length and mild temperatures on winter survival. Physiol. Biochem. Zool.

